# Pregnancy reduces the perception of anxiety

**DOI:** 10.1038/s41598-017-07985-0

**Published:** 2017-08-23

**Authors:** Katrin T. Lübke, Anne Busch, Matthias Hoenen, Benoist Schaal, Bettina M. Pause

**Affiliations:** 1 Department of Experimental Psychology, Heinrich-Heine-University Düsseldorf, D-40225 Düsseldorf, Germany; 2 Centre des Sciences du Goût, CNRS/Université de Bourgogne (UMR 6265), F-21000 Dijon, France

## Abstract

In humans, stress can be contagiously transmitted via chemosignals on a subconscious level. This study investigates how pregnancy affects neural responses to anxiety chemosignals. Using cotton pads, 28 men donated axillary sweat immediately before an academic examination (anxiety sweat) and during ergometer training (control). Via a constant-flow olfactometer, samples were presented (oddball paradigm) to 12 non-pregnant (NP) women, 14 women in their first (T1), and 18 in their third (T3) trimester of pregnancy. Chemosensory event-related potentials and current source densities (CSD) were analysed (60 electrode setup). Compared to NP-women, pregnant women display diminished evaluative processing of the sweat samples (targets; P3-1/ P3-2 amplitudes) and delayed evaluative processing of the anxiety sweat (targets; P3-2 latency). T3-women show attenuated early processing (targets; N1 amplitude) compared to NP-women, and reduced evaluative processing compared to T1-women (standards; P3-2 amplitude). CSDs (P3-1/ P3-2 latency ranges) reveal that T1- and T3-women show an atypical activation distribution to anxiety sweat. Most participants were unable to detect the sweat samples (anxiety sweat: 79.5%, sport sweat 88.6%). The results demonstrate that the processing of anxiety chemosignals progressively vanishes during pregnancy. This effect is likely to occur without any cognitive control.

## Introduction

Women’s socio-emotional perception changes over the course of pregnancy. Pregnant women become increasingly accurate in encoding negative facial expressions^1^, show a marked attentional bias towards fearful faces^2, 3^, and a neural hypervigilance towards angry faces^4^. It is typically argued that such changes reflect a heightened sensitivity to social danger cues in pregnant women. Evolutionary theories suggest that such increased responsiveness to potential threat results from the heightened need for maternal precaution during the vulnerable period of pregnancy^5^. In contrast, stress responses appear largely dampened in pregnancy: Pregnant women have been shown to display attenuated physiological responses to psychological and physiological stressors^6, 7^ in terms of diminished autonomic responses^8–10^ and a reduced cortisol response^11^. Moreover, the stress response of pregnant women shows a progressive decline with advancing pregnancy^12, 13^. While a heightened ability to detect social danger cues may well be advantageous for pregnant women, a stress response to such cues might be fatal: Psychological distress experienced during pregnancy is closely related to relatively short gestational length and preterm delivery^12, 14–16^, as well as adverse neurodevelopmental and health outcome in the offspring^17, 18^, a gradual decrease of stress sensitivity with advancing pregnancy^6, 7^ is discussed as a mechanism protecting the unborn infant.

Among social signals, and in stark contrast to facial affect, chemosensory cues have the unique property of being “honest signals”, as neither their production, nor their release or their informative content are subject to conscious manipulation. Also, the impact of social signals such as fearful faces is subject to individual interpretation and appraisal^19^. In contrast, the ability to regulate emotions elicited by odours appears strongly limited^20^. Indeed, activation in empathy-related brain areas in response to chemosensory anxiety signals^21^ suggests that these signals are contagious. This emotional contagion hypothesis is confirmed by the findings that fear chemosignals induce fearful facial expressions^22^ and increased state anxiety in the perceiving individuals^23^. In line with such emotional contagion, human social perception appears sensitized to detect social signals of threat through chemosensory anxiety signals^19, 24–27^, and adaptive responses such as withdrawal-related motor behaviour are primed^28, 29^, similar to effects demonstrated in rodents^30, 31^. Utilizing the time-sensitive event-related potential technique confirmed that anxiety sweat – albeit being nearly odourless – is processed as a highly significant signal, especially by women^32^.

Taken together, research demonstrates that anxiety sweat is an extremely powerful social signal, not only effectively communicating its emotional content, but facilitating emotional contagion^21–23^, irrespective of its conscious detection^32^. In general, chemosensory social information is mainly processed by the human brain at a subconscious level^33, 34^. The processing of chemosensory anxiety signals thus is a novel and ecologically highly valid paradigm for testing responses to emotionally contagious social cues (“second-hand stress”^25^) in advancing pregnancy. The current study therefore aimed to investigate how early vs. late pregnancy affects the central-nervous processing of human chemosensory anxiety signals. It was hypothesized that during pregnancy, in line with a stress protection mechanism a progressive attenuation of the processing of chemosensory anxiety signals would be observed.

## Materials and Methods

### Participants

Participants were recruited via flyers and announcements in the university, local gynaecologists’ and midwifes’ practices, the university hospital, baby and pregnancy supply stores, and in local newspapers. Of initially 208 applicants, 48 women met the inclusion criteria (see criteria below). Data of another four women were excluded from analyses due to technical issues during EEG recording (n = 2) and pronounced EEG artefacts (n = 2, see data reduction). The final sample thus consisted of 44 right-handed women (Annett Handedness Questionnaire^35^). As axillary sweat production is in part genetically determined, and the respective allelic profiles vary with ethnos^36^, only sweat donors of European origin were included, and accordingly, all participating women were required to be of European descent. Participants were free of any mental disease (Structured Clinical Interview for the Diagnostic and Statistical Manual for Mental Disorders IV^37^). In addition, no participant reported to suffer from any neurological, endocrine or immunological condition, or diseases related to the upper respiratory tract, and none of them reported smoking cigarettes on a regular basis. A brief screening for general hyposmia was introduced, with participants being required to detect phenyl-ethyl alcohol (99%, Fluka, Germany, 1:200 (*v/v*) diluted in 1,2-propanediol) present in one of three bottles in two consecutive trials, with the remaining two bottles containing the same volume of solvent. Phenyl-ethyl alcohol was chosen as test odorant for general hyposmia since it is considered a purely olfactory odour used as a standard in olfactory sensitivity testing^38^, and to date no case of specific anosmia to phenyl-ethyl alcohol has been reported^39^. The brief screening test revealed no suspicion of general hyposmia in any participant.

Of the final 44 participants (mean age = 29.2 years, SD = 5.7 years, age range = 19–44 years), 12 women were neither pregnant nor planning to become pregnant (NP), 14 were in their first (T1, average gestational week = 11.2, SD = 1.4 weeks), and 18 were in their third trimester of pregnancy (T3, average gestational week = 29.4, SD = 4.3 weeks). Pregnant women reported having a normal course of pregnancy (German scale of attitudes toward pregnancy^40^). The majority of the pregnant women in each group were primiparous (T1: n = 10, T3: n = 11). The level of social anxiety (as measured via the Social Interaction Anxiety Scale, SIAS^41^) did not differ between experimental groups (M = 10.18, SD = 5.85; p > 0.25), nor did their general happiness (M = 2.02, SD = 0.90, indicating a positive mood) or arousal level (M = 5.02, SD = 1.25, indicating a medium arousal, all p_s_ > 0.06; Self-Assessment Manikin, SAM^42^; introduced as a trait measure). The non-pregnant women (n = 7 taking oral contraceptives) reported having a regular menstrual cycle (M = 28.2, SD = 1.7 days). All participants gave written informed consent, and were paid for their participation. The study was carried out in accordance with the Declaration of Helsinki, and was approved by the ethical committee of the Medical Faculty of the University of Kiel.

### Chemosensory stimuli and stimulus presentation

**Figure 1:**
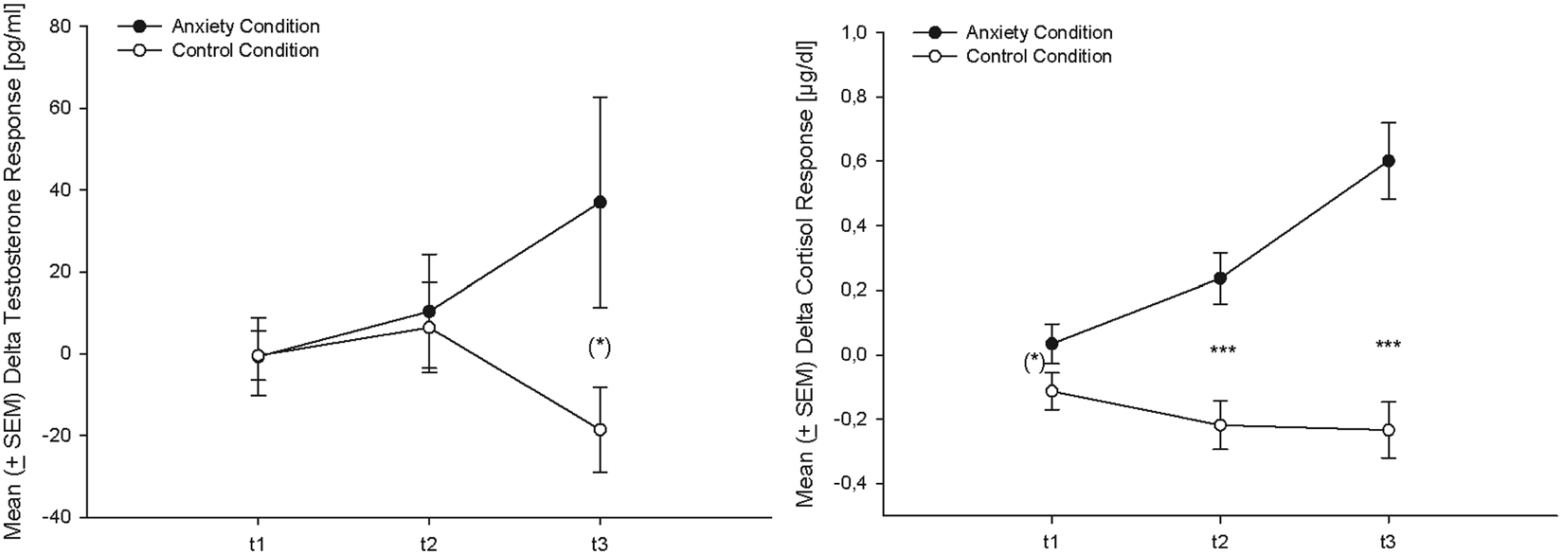
Testosterone (left, n = 17) and cortisol (right, n = 18) change-scores (in reference to baseline: Mean ± SEM) at the three time points (t1 = 30 minutes prior to the oral examination/ following the first bicycling set, t2 = immediately before the oral examination/ following the third bicycling set, t3 = immediately after the oral examination/ by the end of the ergometer session, time points separated by 30 min each), (*)p < 0.10, ***p < 0.001 (according to t-tests).

Axillary sweat serving as anxiety signal was collected from 28 male students for a duration of 1 hour while awaiting their final oral examination at the university. The chemosensory control stimulus was composed of sweat samples from the same individuals while participating in light ergometer training (3 bicycling sets of 10 minutes at a constant heart rate of 110 bpm, separated by breaks of ten minutes; total duration of 1 hour including instructions). Each donor thus participated in two sessions on different days, with each session being scheduled at the same hour of the day. On average, both sessions were scheduled 2.2 (SD = 0.6) days apart from each other. The donors felt less happy (state SAM: valence, t(27) = 6.14, p < 0.001, see Table 1) and more submissive (state SAM: dominance, t(27) = 5.17, p < 0.001) during the anxiety compared to the sport condition. Arousal did not differ between conditions (state SAM: arousal, p = 0.14, all p-values are Bonferroni corrected). Both salivary cortisol (n = 18) and testosterone (n = 17) increased during the anxiety condition, and decreased during the ergometer condition (condition x time, cortisol: F(2, 34) = 14.81, p < 0.001, testosterone: F(2, 32) = 3.98, p = 0.04, see Fig. 1). Prior to presentation, the sweat samples were pooled across donors with respect to the chemosensory condition and divided into portions of 0.4 g. The sweat donors and the sampling procedure are described elsewhere in more detail^21^.

**Table 1:** Emotions expressed by the sweat donors (n = 28) using the Self-Assessment Manikin (state measure).

Dimension	Anxiety Condition		Sport Condition	
	M	SD	M	SD
Valence	0.96***	1.50	3.29	1.33
Arousal	6.36	1.31	5.68	1.42
Dominance	4.61***	1.37	6.46	1.40

Valence: range −4 to +4, Arousal: range 1 to 9, Dominance: range 1 to 9, ***p < 0.001 (difference between anxiety and sport condition, Bonferroni corrected).

The sweat samples were presented according to the method described by Kobal^43^, using a constant air-flow (100 ml/s; stimulus duration = 0.5 s), six channel olfactometer (OM6b, Burghart, Wedel, Germany). Both nostrils were stimulated simultaneously, and accordingly, both air streams were controlled by separate mass-flow meters. In the olfactometer, the glass tubes containing the stimuli (0.4 g of each stimulus per nostril) were stored in a warm-water chamber, and the stimuli were delivered to the participants through a Teflon tube. The temperature of the air flow at the exit of the olfactometer was 37 °C and the relative humidity was set above 80%. White noise of 85 dB (A) was presented binaurally over earplugs (Etymotic Research, ER3-14A) in order to prevent the participants from hearing the switching valves of the olfactometer.

### Odour detection and ratings

In order to infer whether the sweat samples were presented mostly above or below the level of conscious perception, it was determined in how far the participants were able to consciously detect the odour of anxiety and sport sweat, respectively. The participants had to select the most intense stimulus from a series of three (presented for 0.5 s each), with the remaining two blanks consisting of pure, unused cotton pads. This procedure was carried out twice for each sweat sample prior to the EEG recording. Participants who failed once to detect the relevant sweat sample were considered not to be able to smell the respective odour. Moreover, in order for the participants to remain attentive during EEG recording, they were instructed to silently count the instances during which they believed to perceive an odour during the EEG recording (max n = 100). Using the state SAM, the participants described their emotions while they were exposed to each sweat sample for 3 s. In each instance the sweat samples were presented via the olfactometer.

### Procedure

All participants were tested individually and were on their own during EEG recording. Prior to the EEG recording, participants practiced the velopharyngeal closure technique^44^ and were instructed to maintain this breathing technique throughout the EEG recording. This breathing technique was introduced in order to ensure valid detection of early CSERP components^44^. The participants were further instructed to avoid eye movements. The EEG was recorded during an oddball paradigm consisting of two blocks of 100 trials each (25 deviant chemosensory stimuli in a train of 75 standard stimuli). Both types of stimuli were presented in pseudo-randomized order (with the first three trials being standards) for 0.5 s with an inter-stimulus interval (ISI) of 9 s. In each of the two blocks, the standard stimulus was either the anxiety or the sport stimulus, with the order of these blocks counterbalanced across participants. The blocks were separated by a break of 5 minutes, resulting in a total of approximately 35 minutes duration of the EEG procedure. The mean duration of a session was 2.4 (SD = 0.4) hours, and the room temperature was kept between 19–21 °C.

### Data Recording and Reduction

Ongoing EEG was recorded from 60 scalp locations (in reference to the left earlobe) with Ag/AgCl electrodes (inner diameter 6 mm), using an electrode cap (EasyCap GmbH, Germany). Two additional electrodes were placed near the right eye (3 cm above, inside the vertical pupil axis and 1.5 cm below, outside the vertical pupil axis) for the recording of vertical and horizontal eye movements. The impedance of the electrodes was always below 11 kΩ.

**Figure 2:**
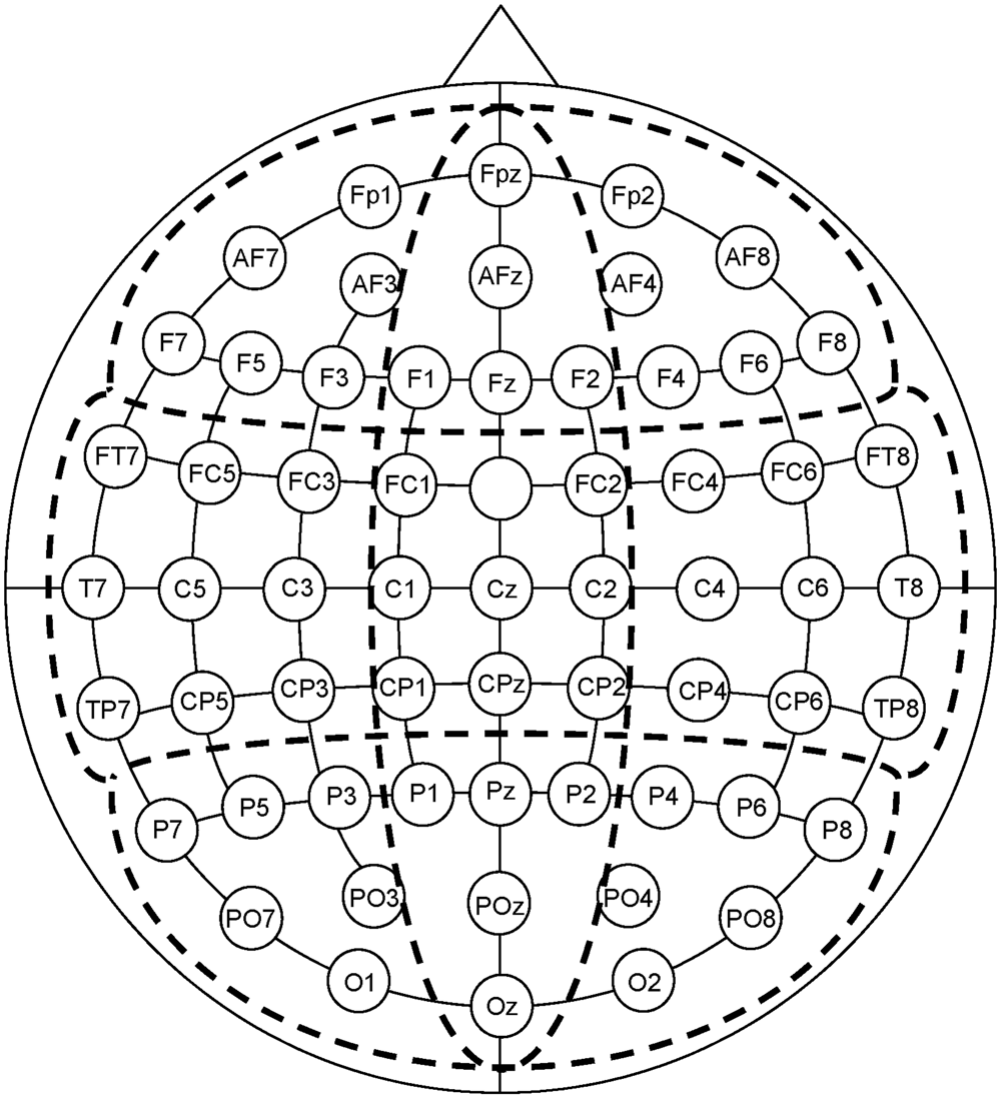
Layout of the nine electrode pools. Sagittal line: anterior (a), central (c), posterior (p); transversal line: left (l), midline (m), right (r); sagittal by transversal: al: Fp1, AF7, AF3, F7, F5, F3; am: FPz, AFz, F1, Fz, F2; ar: Fp2, AF4, AF8, F4, F6, F8; cl: FT7, FC5, FC3, T7, C5, C3, TP7, CP5, CP3; cm: FC1, FC2, C1, Cz, C2, CP1, CPz, CP2; cr: FC4, FC6, FT8, C4, C6, T8, CP4, CP6, TP8; pl: P7, P5, P3, PO7, PO3, O1; pm: P1, Pz, P2, POz, Oz; pr: P4, P6, P8, PO4, PO8, O2. The ground electrode was placed at Fcz.

The physiological data were recorded, amplified, and filtered with the Aquire software (Version 4.2, NeuroScan Inc., Virginia, USA) using a sampling rate of 200 Hz, a low-pass filter of 40 Hz (24 dB/ octave) and a 50 Hz notch filter. Offline, EEG signals were rereferenced to linked ear lobes, baseline corrected (0–1000 ms before stimulus onset), and high pass filtered (0.2 Hz, 24 dB/ octave). The data were then corrected for eye movements^45^. In addition, trials contaminated by any further artefacts (amplitudes exceeding +50 µV or below −50 µV) were eliminated from the analysis. Subsequently, a zero phase shift digital low pass filter (Butterworth-filter, 7 Hz, 24 dB/ octave) was applied. The 60 scalp electrode positions were subdivided into nine areas, and a mean ERP for each area was calculated by averaging adjacent electrodes in anterior, central, and posterior areas for the left and right hemisphere as well as for midline electrodes (see Fig. 2). In relation to the baseline period, four separate peaks were differentiated within predefined latency windows (N1: 250–500 ms, P2: 500–700 ms, P3-1: 700–900 ms, P3-2: 900–1100 ms). While the N1 and P2 reflect early, preattentive processing mainly driven by stimulus features, the P3-1, showing a fronto-central dominance, is regarded as a “novelty P3”, and the P3-2 with its parieto-central dominance reflects the common P3, occurring independently of the specific modality and in response to significant stimuli^46, 47^. Evaluative decoding processes, as reflected by the P3-1 and P3-2, are prominent in response to rare, deviant stimuli, while responses to standard stimuli are rather driven by automatic encoding processes^46^. In case of the CSERP in response to standard stimuli, habituation processes resulted in a reduction of amplitude size across groups, especially evident within the small high-frequency peaks of the N1, P2, and P3-1. This effect rendered a valid peak detection of these components in response to standard stimuli impossible. Thus, amplitudes and latencies of all detected peaks in response to deviant stimuli were analysed, while only the P3-2 in response to standard stimuli underwent statistical analysis.

### Data Analysis

Amplitudes and latencies of the CSERP components were analysed by means of a four-way ANOVA, including the factors Group [non-pregnant women (NP), pregnant women in the first trimester of pregnancy (T1), pregnant women on the third trimester of pregnancy (T3), Sweat Sample (anxiety sweat, sport sweat), Sagittal (anterior, central, posterior), and Transversal (left, midline, right). In order to focus effects of the chemosensory stimuli within each of the three groups, separate 3-way ANOVAs containing the factors Sweat Sample (anxiety sweat, sport sweat), Sagittal (anterior, central, posterior), and Transversal (left, midline, right) were conducted for each group. Subsequently, nested effects were isolated and calculated^48^. An alpha level of p < 0.05 was used for all statistical tests. Huynh-Feldt corrected degrees of freedom were calculated, and corrected p-values are reported. Current Source Density (CSD) maps were calculated using a spherical spline model^49^ (order of splines: m = 4, maximal degree of Legendre polynomials = 20).

### Data Availability

The datasets generated and analysed during the current study are available from the corresponding author on reasonable request.

## Results

### Odour detection

Overall, 79.5% of the participants were not able to detect the odour of anxiety sweat, and 88.6% did not detect the odour of sport sweat. The proportion of individuals able to detect a given odour did not differ with respect to the chemosensory sample (χ^2^ (1) = 1.23, p = 0.27, McNemar-Test) or with respect to pregnancy status (anxiety sweat: p = 0.29, sport sweat: p = 0.39; Fisher’s Exact Test).

### CSERPs

#### Deviant Stimuli

Amplitudes: to be in line with Latencies: Both pregnancy and type of sweat affected amplitudes indicative of early automatic and late evaluative processing. With advancing pregnancy, the early processing of the sweat samples was particularly attenuated, as women in their third, but not women in their first trimester of pregnancy showed reduced amplitudes of the N1 component (Group: F(2, 41) = 4.33, p = 0.02, η^2^_p_ = 0.175; T3 vs. NP: t(28) = 2.90, p < 0.01; see Tables 2 and 3). Furthermore, pregnant women in general displayed smaller P3-1 (Group: F(2, 41) = 5.14, p = 0.01, η^2^_p_ = 0.200; T1 vs. NP: t(24) = 2.20, p = 0.04; T3 vs. NP: t(28) = 3.05, p < 0.01) and P3-2 amplitudes (Group: F(2, 41) = 4.71, p = 0.01, η^2^_p_ = 0.187; T1 vs. NP: t(24) = 2.24, p = 0.04; T3 vs. NP: t(28) = 2.70, p = 0.01) than non-pregnant women (see Table 2 for descriptive statistics).

**Table 2:** CSERP-amplitudes of non-pregnant women, women in their first, and women in their third trimester of pregnancy.

Stimuli		NP	T1	T3	Main effect “Group”
Deviant	N1	−1.31 (±0.34)	−0.87 (±0.28)	−0.21 (±0.21)	p = 0.02
Deviant	P3-1	2.87 (±0.60)	1.30 (±0.42)	1.08 (±0.27)	p = 0.01
Deviant	P3-2	3.06 (±0.66)	1.44 (±0.35)	1.18 (±0.36)	p = 0.01
Standard	P3-2	1.82 (±0.39)	1.19 (±0.27)	0.81 (±0.19)	p = 0.04

Notes: Amplitudes are given as mean values (±SEM) in µV; NP = non-pregnant women, T1 = women in their first trimester of pregnancy, T3 = women in their third trimester of pregnancy.

**Table 3:** CSERP in response to deviant and standard stimuli: Overall analysis of variance of amplitudes and latencies. Significant main effects, interactions, and single comparisons.

Stimuli	Detected peaks	Sweat Sample	Group	Group by Sweat Sample	Group by Sagittal	Group by Transversal	Sweat Sample by Transversal	Group by Sagittal by Transversal
Deviant	N1 amplitude		T3 < NP**					
Deviant	P3-1 amplitude		T1 < NP*			T1 < NP in m*, r*		
			T3 < NP**					
						T3 < NP in l**, m**, r*		
Deviant	P3-2 amplitude	anxiety > sport*	T1 < NP*		T1 < NP in c*	T1 < NP in m*	anxiety > sport in m*	T1 < NP in cm*, pm*
			T3 < NP**					
					T3 < NP in c**, p**	T3 < NP in l*, m**, r*		T3 < NP in cl**, cm**, pl*, pm**, pr**
Deviant	P3-2 latency	sport > anxiety**		T1 > NP in anxiety sweat*				
				T3 > NP in anxiety sweat***				
Standard	P3-2 amplitude		T3 < NP*		T3 < NP in c*, p*	T3 < NP in m**, r**		T3 < NP in cm**, cr*, pl*, pm**, pr**
					T3 < T1 in p*			
								T3 < T1 in cr*, pr*

Notes: anxiety = anxiety sweat, sport = sport sweat; NP = non-pregnant women, T1 = women in their first trimester of pregnancy, T3 = women in their third trimester of pregnancy; Sagittal line: a = anterior, c = central, p = posterior; Transversal line: l = left, m = midline, r = right, *p ≤ 0.05, **p ≤ 0.01, ***p ≤ 0.001.

The difference between women early in pregnancy and non-pregnant women regarding the P3-1 was especially evident in midline and right electrode pools, while smaller amplitudes of late pregnant women compared to non-pregnant women were evident in all electrode pools (left, midline, right; Group x Transversal: F(4, 82) = 4.48, p < 0.01; see Table 3). Smaller P3-2 amplitudes in early pregnant women compared to non-pregnant women were observable specifically in central-midline and posterior-midline electrode pools where the P3-2 is dominant, while the difference between late pregnant and non-pregnant women was evident in central-left, central-midline, and across all posterior electrode pools (posterior-left, posterior-midline, posterior-right; Group x Sagittal x Transversal: F(8, 164) = 2.33, p = 0.03; see Table 3, also see Table 3 for further interactions mirroring the effects reported here).

Late stimulus processing further was affected by the specific type of sweat presented, as across all participants, P3-2 amplitudes were larger in response to anxiety compared to sport sweat (Sweat Sample: F(1, 41) = 5.07, p = 0.03, η^2^_p_ = 0.110), especially in midline electrode positions (Sweat Sample x Transversal: F(2, 82) = 3.57, p = 0.03).

**Figure 3:**
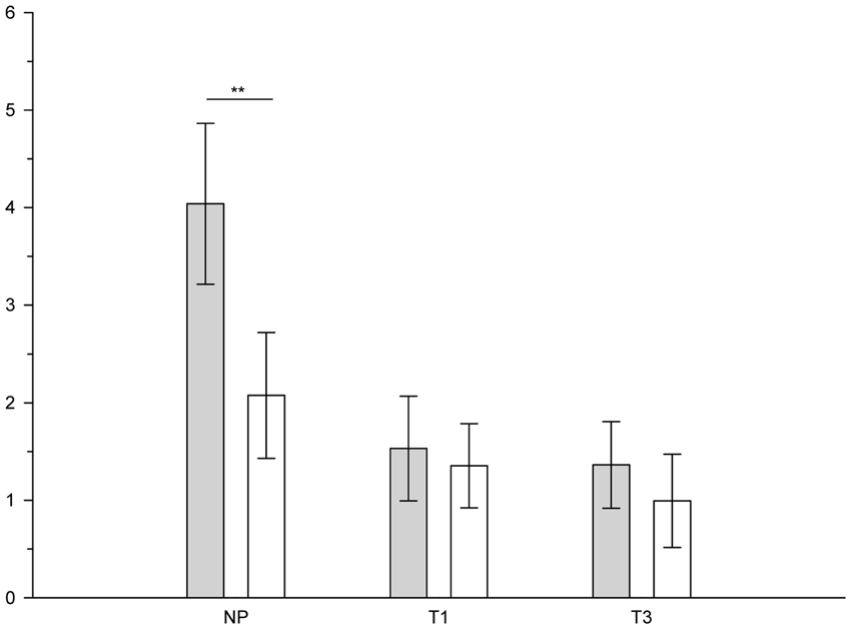
Mean (±SEM) P3-2 amplitudes (µV) in response to anxiety sweat (grey bars) and sport sweat (white bars) deviant stimuli of non-pregnant women (NP), pregnant women in the first (T1), and pregnant women in the third trimester (T3), **p ≤ 0.01.

Analysing the effects of sweat type separately in pregnant and non-pregnant women revealed that pregnant women did not show the particular differential P3-2 response of larger amplitudes in response to anxiety compared to sport sweat, while non-pregnant women did (Sweat Sample: F(1, 11) = 8.77, p = 0.01, η^2^_p_ = 0.444, see Fig. 3).

**Figure 4:**
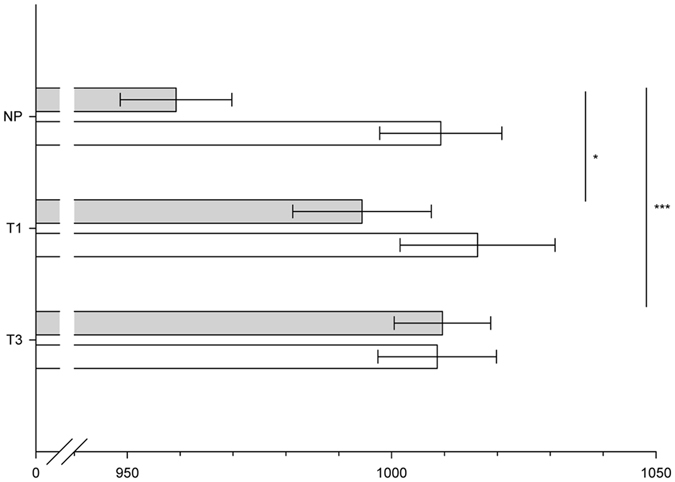
Mean (±SEM) P3-2 latencies (ms) in response to anxiety sweat (grey bars) and sport sweat (white bars) deviant stimuli of non-pregnant women (NP), pregnant women in the first (T1), and pregnant women in the third trimester (T3), *p ≤ 0.05, ***p ≤ 0.001.

Latencies: Similar to the amplitudes, the latencies of the CSERP were affected by pregnancy status and sweat type. Regarding late evaluative processing it appeared that P3-2 latencies were shorter in response to anxiety compared to sport sweat across all participants (Sweat Sample: F(1, 41) = 7.03, p = 0.01, η^2^_p_ = 0.146). Pregnancy status specifically affected responses to anxiety sweat, as pregnant women showed prolonged P3-2 latencies in comparison to non-pregnant women (Group x Sweat Sample: F (2, 41) = 2.77, p = 0.08, η^2^_p_ = 0.119; NP vs. T1 within anxiety sweat: t(24) = 2.04, p = 0.05, NP vs. T3 within anxiety sweat: t(28) = 3.57, p = 0.001, see Fig. 4).

**Figure 5:**
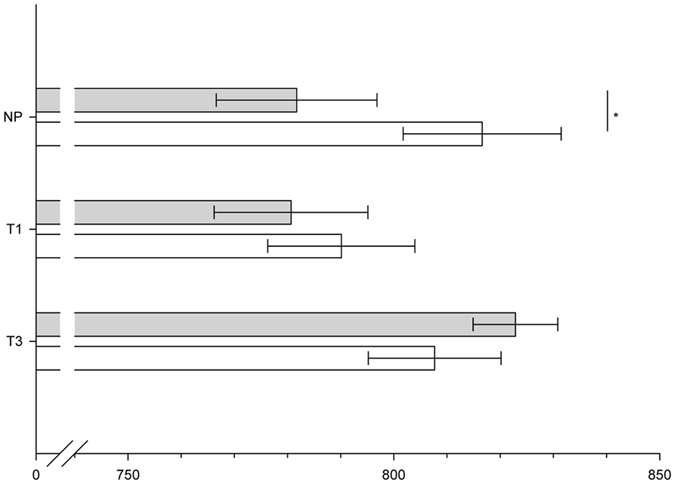
Mean (±SEM) P3-1 latencies (ms) in response to anxiety sweat (grey bars) and sport sweat (white bars) deviant stimuli of non-pregnant women (NP), pregnant women in the first (T1), and pregnant women in the third trimester (T3), *p < 0.05.

When analysing pregnant and non-pregnant women’s responses separately, effects of sweat type were evident regarding early as well as late processing. Regarding early automatic processing, non-pregnant, but not pregnant women responded with shorter P3-1 latencies (Sweat Sample: F(1, 11) = 6.12, p = 0.03, η^2^_p_ = 0.358) to anxiety sweat as compared to sport sweat (see Fig. 5). Moreover, similar to the pattern observed within the amplitudes, pregnant women did not show the particular differential P3-2 response of shorter latencies in response to anxiety compared to sport sweat, while non-pregnant women did (Sweat Sample: F(1, 11) = 17.17, p = 0.02, η^2^_p_ = 0.610).

#### Standard Stimuli

The amplitudes of the P3-2 in response to standard stimuli were largely affected by pregnancy progression as they showed a gradual decline over the course of pregnancy. Only women in late, but not women in early pregnancy showed smaller P3-2 amplitudes than non-pregnant women (Group: F(2, 41) = 3.45, p = 0.04, η^2^_p_ = 0.144; T3 vs. NP: t(28) = 2.59, p = 0.02, see Table 2 for descriptive statistics). These reduced amplitudes of late pregnant women were especially evident in central-midline, central-right, and across all posterior electrode pools (Group x Sagittal x Transversal; F(8, 164) = 3.65, p = 0.001; see Table 3). Moreover, across central-right and posterior-right scalp regions, women in late pregnancy even showed reduced amplitudes when compared to women in early pregnancy (see Table 3). P3-2 latencies in response to standard stimuli were unaffected by sweat type and pregnancy status.

### Current source densities

**Figure 6:**
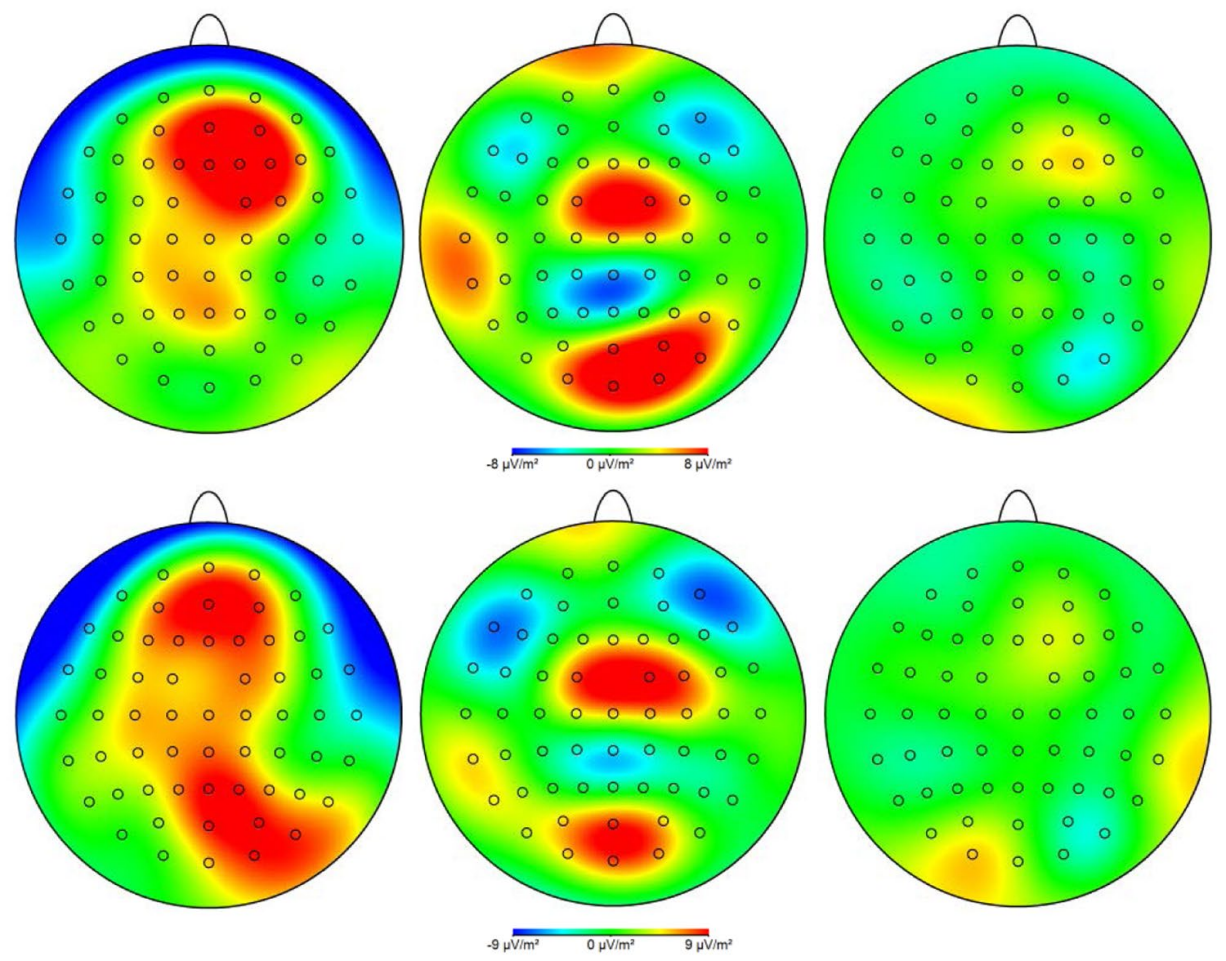
Neural processing of anxiety sweat (deviant stimuli) at the time of maximum P3-1 (upper row) and P3-2 amplitude (lower row) of non-pregnant women (left column), first-trimester pregnant women (middle column) and third-trimester pregnant women (right column), plotted as current source density (CSD) maps. Blue colours represent a weaker magnitude (neuronal sinks) and red colours represent a stronger magnitude of CSD (neuronal sources).

Mirroring the CSERPS results, the neuronal activation in response to anxiety sweat in pregnant women (T1, T3) appears less intense, regarding extension and strength of neuronal sources, than in non-pregnant women (see Fig. 6). In detail, within the time-frame of the maximal P3-1 amplitude, non-pregnant women show a rather widespread distribution of neuronal sources with pronounced fronto-medial dominance. Within the P3-2 time-frame, the maximum of neuronal sources is shifted to parieto-medial regions. The extension of neuronal sources of women in early pregnancy (T1) is comparably smaller, and appears with a centro-medial and a parieto-medial focus in both the P3-1 and the P3-2 time-range. Women in late pregnancy (T3) show barely detectable neural responses to the sweat derived social signals in either time-frame.

### Ratings and odour count

The participating women described the emotions induced by being exposed to the sweat samples as rather neutral (SAM, valence: M = 0.15, SD = 1.34), of medium arousal (SAM, arousal: M = 4.30, SD = 1.40), and medium dominance (SAM, M = 5.08, SD = 1.23). Descriptions of valence, arousal, and dominance did not differ with respect to pregnancy status or the presented sweat sample (all p_s_ > 0.50).

However, the three groups of women differed in the number of odours they perceived during EEG recording (F(2, 41) = 3.22, p = 0.05), with non-pregnant women reporting on average 37.4 (SD = 35.4) counted stimuli, women in their first trimester of pregnancy reporting 29.6 (SD = 19.8), and women in their third trimester of pregnancy reporting 15.0 (SD = 19.0) stimuli.

## Discussion

The current study is the first to show diminished automatic and evaluative brain responses in pregnant women to the exceptionally significant social signals deriving from human anxiety sweat. In early pregnancy (first trimester) brain response patterns are in line with a cortical dysregulation, while in late pregnancy (third trimester) brain responses to chemosensory anxiety verged on being non-detectable. Large effect sizes show that these effects are exceptionally substantial. The current results thus contrast reports of pregnant women being overly sensitive to social signals of threat or harm. Rather, they can be interpreted along the line of a protective mechanism at work during pregnancy, preventing pregnant women from automatic emotional contagion via chemical signals.

In more detail, non-pregnant women display enhanced (P3-2 amplitudes) and accelerated evaluative (P3-2 latencies) as well as accelerated stimulus-driven automatic processing (P3-1 latencies) of anxiety sweat compared to sport sweat. These results corroborate earlier findings of enhanced processing of significant chemosensory social signals in human sweat^32, 50^. The accelerated occurrence of the P3-1 is most probably a consequence of the processing of biologically relevant signals contained in axillary sweat emanating from individuals who experience anxiety. These signals obviously induce the quick attentional shift indicated by the P3-1^51^. The larger P3-2 amplitude and shorter P3-2 latency in response to anxiety compared to sport sweat probably relate to the mobilization of a higher amount of neuronal resources that are recruited quite quickly. This would be well in line with imaging studies showing that more neuronal resources are involved in the processing of emotional chemosignals compared to control sweat samples^21, 52^. Importantly, even though women taking oral contraceptives were included in the sample of non-pregnant women, which has been reported to affect chemosensory perception^53^, all of them showed reliably detectable neural responses to the chemosensory stimuli. In total, the pronounced evaluative processing implies anxiety sweat to contain subjectively highly relevant (alarm) signals^32, 54^, possibly activating preparedness for a subsequent adaptive response. Interestingly, and in line with earlier work^21, 32, 52^, the majority of participating women was not able at all to detect the odour of the sweat samples in a forced choice test. Thus, to a vast majority of women, the sweat samples were presented below the threshold of conscious awareness. Correspondingly, ratings of experienced valence, arousal, and dominance during sweat sample presentation were in a medium range, and did not differ between the sweat samples. The neuronal processing of chemosensory anxiety signals thus is considered not to be consciously mediated.

In contrast to non-pregnant women, in pregnant women (first and third trimester) enhanced or accelerated evaluative (P3-2 amplitude, P3-2 latency) processing of the chemosensory alarm signal is strikingly absent. Both groups of pregnant women even show a delay in the EEG correlate of evaluative responses (P3-2 latencies) to anxiety sweat in direct comparison to non-pregnant women. In line with altered attentional processing, non-pregnant women reported significantly higher number of odours perceived during EEG recording than both groups of pregnant women, and women early in pregnancy still reported higher numbers than women in late pregnancy. The results of the odour counting show a different pattern than those of the forced choice detection test, however, the detection test mainly involves bottom-up processing (sensitivity), while the odour counting during 35 minutes of EEG recording is affected by bottom-up as well as top-down processes, such as attention (for discussion see^55^).

Current source density analyses further revealed that the attenuated processing of the chemosensory signals was not only progressive over the course of pregnancy, but also showed qualitative changes: Women in the first trimester still showed neuronal responses to anxiety sweat within the P3-1 and P3-2 latency ranges, but with an atypical spatial distribution indicating a cortical dysregulation. Women in the third trimester of pregnancy did not show any detectable neuronal sources during late processing when presented with anxiety sweat. Thus, the decoding of the alarm signal’s psychobiological relevance together with its automatic attention-capturing features is quantitatively and qualitatively altered in early vs. late pregnancy.

Interestingly, even authors generally reporting a “hypervigilance” of pregnant women towards social signals incidentally report results similar to those of the current study. Women in middle and late pregnancy have been shown to display a dysregulation of prefrontal cortical activity during the processing of fearful faces which seems to increase over the course of pregnancy^3^, and women in late pregnancy display diminished and delayed processing of angry facial expressions (P3 component of the event-related potential), in line with reduced attentional capacities^4^.

A progressive alteration in the processing of chemosensory social signals is further evident as women late in pregnancy, in contrast to women early in pregnancy, display reduced N1-amplitudes, which reflect the earliest stage of chemosensory processing. As this variation cannot be explained by differences in perceived intensity, it is proposed that women late in pregnancy directed comparably less attentional resources towards the chemosensory signal^47^. Another indicator of a progressive change in the processing of chemosensory social signals over the course of pregnancy is, that while both groups of pregnant women display reduced P3-2 amplitudes compared to non-pregnant women, this reduction is most wide-spread in women late in their pregnancy: Women early in pregnancy show reduced P3-2 amplitudes mainly above scalp areas where the P3-2 is most dominant (central-midline and posterior-midline)^46^, while women late in pregnancy show reduced P3-2 amplitudes across all but one central and posterior areas (central-left, central-midline, posterior-left, posterior-midline, posterior-right). Moreover, even in response to standard stimuli, women in late pregnancy feature reduced P3-2 amplitudes compared to women in early pregnancy, again supporting qualitative changes in chemosensory social signal processing over the course of pregnancy.

In order to investigate whether the processing of social chemosignals linearly changes with the gestational week, an exploratory analysis was conducted, revealing that, by trend, the later the female participants were in their pregnancy the more reduced the N1 amplitude (r = 0.298, p = 0.10), and the more delayed the P3-1 component appeared (r = 0.315, p = 0.08). Regarding the processing of anxiety sweat in particular, the P3-1 amplitude showed a progressive reduction with advancing pregnancy (r = 0.430, p = 0.01). These effects might be interpreted along the line of at least some changes in the responsiveness to chemosensory social signals occurring during pregnancy showing a linear relationship with gestational age. However, the design of the current study is factorial in nature, and data of women in the second trimester of pregnancy are missing, thus these correlations need be interpreted with caution.

Within the current study, the participating women were presented with male anxiety sweat only. However, it has been proposed that men and women are equally capable of producing chemosignals effectively communicating anxiety. A study adopting a design similar to the current one showed neural responses to anxiety sweat to be unaffected by the sex of the sweat donors^32^. Thus, it is assumed that the current results are generalizable to female anxiety sweat as well.

Taken together, the current results clearly demonstrate a decreasing and altering response to chemosensory anxiety signals over the course of pregnancy. Anxiety sweat is most probably able to transmit anxiety to perceiving individuals, that is, to foster emotional contagion^56^. An augmented startle response^28^ in the context of chemosensory anxiety signals as well as the recruitment of empathy-relevant brain areas during anxiety sweat processing^21^ are indicative of such contagious processes. Importantly, this chemosensory contagion happens without conscious awareness, as across studies, most participants do not report any conscious percept while being exposed to anxiety sweat^21, 32, 52^. Thus, anxiety contagion via chemical signals most probably happens directly and inevitably, and cannot be attentively controlled or regulated by the perceiving individual. The fact that pregnant women do not specifically respond to anxiety sweat could be related to a diminished stress response emerging during pregnancy^6, 7^: Given the substantial risks for motherly and infant health resulting from maternal intrapartum distress^57^, a decreasing and altering processing of chemosensory anxiety signals during pregnancy probably reflects a basic mechanism protecting the unborn infant against maternal stress deriving from automatic emotional contagion. It is therefore not surprising that pregnant women’s responses to visual social signals^1–4^ contrast with those to chemosensory social signals apparent in the current study. Facial expressions do not necessarily lead to emotional contagion, but are subject to individual interpretation and appraisal^19^, while chemosensory social signals automatically exert their effects without conscious evaluation. Gestational age is a rarely studied factor in research on stress responses in pregnancy, however, some studies show that pregnant women experience stressful events as more stressful early in pregnancy compared to late pregnancy^12, 13^. This pattern shows close similarity to what was observed in the current study, with first trimester women still responding somewhat to the chemosensory signals, but third trimester women showing virtually no response anymore.

Regarding potential underlying mechanisms, a pregnancy-related change of olfactory processing is unlikely to explain the current results. Despite pregnant women often reporting heightened olfactory reactivity or a disturbed sense of smell, such reports do not hold up to empirical testing of sensitivity^58^. The changes in central-nervous responsiveness to chemosensory alarm signals might rather relate to the maternal brain undergoing a variety of hormonally mediated alterations^59^. For example, the maternal hypothalamus-pituitary-adrenal (HPA) axis is increasingly downregulated during pregnancy. The resulting reduction in cortisol secretion might involve a diminished responsiveness to social threat signals^60^. On the other hand, an elevated oxytocin level, as present in pregnant women, has been shown to attenuate neural responses to social threat signals^61^. Even the high level of progesterone and estradiol in pregnancy might affect the processing of socio-emotional cues^62^. One potential neural substrate might relate to alterations of prefrontal cortical (PFC) activity. In pregnant women, a decreased selective attention to fearful faces is associated with PFC activation^3^, which in turn might be affected by gonadal hormones and cortisol. As the PFC is a key structure in emotion regulation^63^, it may well be affected by pregnancy-related alterations that dampen stressful experiences for mother and unborn infant.

Importantly, changes to the maternal endocrine system appear progressive over the course of pregnancy^6^, and thus their effects may vary depending on gestational age. Animal research has shown an increasing attenuation of HPA axis responsivity as well as the oxytocin secretory response to physical and emotional stress with progression of pregnancy^64^.

Concluding, the present research demonstrates decreasing as well as qualitatively altering processing of chemosensory anxiety signals during pregnancy. The processing of chemosensory anxiety signals in general provides the basis for the chemosensory transmission of anxiety, facilitating adaptive responses such as defence. The striking unambiguousness of the current data may well result from the unique features of chemosensory anxiety signals on one hand, and of chemosensory perception on the other hand: For one, chemosensory anxiety signals are inherently honest, and carry the pristine information of threat. Second, chemosensory perception itself is omnipresent, as one is unable to “decide” when to perceive a chemosensory signal that is present in ambient air. Thus, in order for a stress protection mechanism to work in case of chemosensory signals, their perception has to be prevented at the earliest possible level of processing in pregnant women, that is, at the level of (pre-attentive) central processing. Such altered responsiveness most probably reflects an evolutionary conserved mechanism protecting pregnant women and their offspring from adverse effects of automatic anxiety contagion via chemical signals that are otherwise ubiquitously functional in humans and across phyla.
